# Material Characterization of Polypropylene and Polystyrene Regarding Molecular Degradation Behavior

**DOI:** 10.3390/ma16175891

**Published:** 2023-08-28

**Authors:** Christoph Schall, Volker Schöppner

**Affiliations:** Kunststofftechnik Paderborn, Paderborn University, 33098 Paderborn, Germany

**Keywords:** material characterization, degradation, stress, loading, viscosity, test stand, deterioration, recycling

## Abstract

During the processing of thermoplastics, polymers are subjected to high stress. As a result of this stress, the polymer chains break, leading to a lower molar mass. This further leads to a lower viscosity of the plastic melt and, eventually, to poorer mechanical properties of the manufactured plastic product. Especially in the context of recycling plastics, this poses a challenge to process technology and product properties. This work aims is to provide a prediction of the material degradation under known stress, so that, for example, a process design that is gentle on the material can be carried out. In order to be able to predict material degradation under a load, a test stand for defined material degradation was designed. The test stand allows for material damaging under a defined temperature, shear rate and residence time. At the same time, the test stand can be used to measure the viscosity, which is used to describe the degradation behavior, since the viscosity correlates with the molar mass. The measured decrease in viscosity under stress can be used to predict material damage under the influencing variables of temperature, shear rate and residence time by means of a test plan and a suitable mathematical description of the measured data. The mathematical description can thus be integrated into simulation environments for plastics processing, so that a simulation of the material degradation can be carried out, if necessary also taking the viscosity reduction into account.

## 1. Introduction

**Figure 1:**
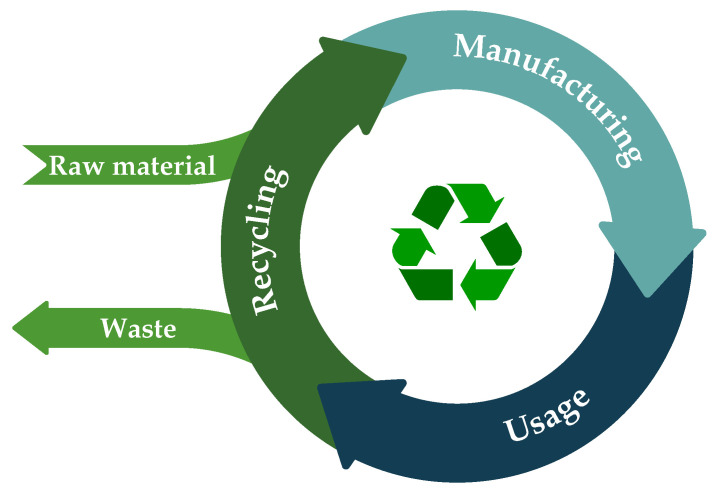
Scheme of a circular economy.

Thermoplastics are one of the most important materials of the 21st century. Through the primary shaping process, continuous products such as pipes, films, sheets and profiles can be produced through the extrusion process, as well as molded parts such as housings and toothbrushes, but also gear wheels and entire dashboards. However, since plastics are largely synthesized from oil and more and more plastic products are ending up as waste, the recycling of plastics is more important than ever before and will continue to grow in importance. As oil is a finite resource, there is a drive towards a circular economy (see Figure 1), which aims to reduce the use of newly synthesized plastic and increase the proportion of reused plastics.

The recycling of thermoplastics implies that the plastic has to be reprocessed many times after its original synthesis. However, each new processing means that a thermoplastic is melted at high temperatures and subjected to high shear rates during processing on an extruder or injection molding machine. This high stress causes the molecular chains to break, the viscosity to drop, the mechanical properties to deteriorate and low-molecular components to be split off, which can lead to odor formation and discoloration of the plastic. For this reason, it makes sense to design the manufacturing processes in such a way that the material damage can be kept low, so that the number of processing cycles of the plastics can be increased and the properties of the manufactured plastic products can be maintained as far as possible. However, this is not possible with the current state of research due to the lack of a possibility to predict the expected material degradation during processing.

## 2. State of Research

The consideration of the molecular degradation of plastics during processing, for example by extrusion or injection molding, is not state-of-the-art. However, the fact that the molar mass of molten plastics decreases under high stress (through shearing and temperature) is known and was already investigated in the 1960s [1,2,3,4,5,6,7].

**Figure 2:**
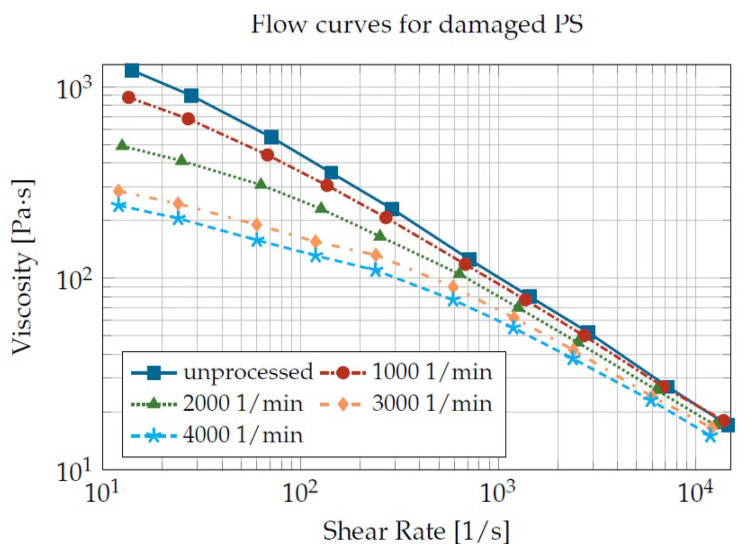
Exemplary flow curves for a damaged polystyrene according to [8]. Damage due to extrusion on a co-rotating ⌀ 15 mm twin-screw extruder.

The reduction in the molar mass means that the molecular chains of the polymer are decreasing in length. The shortening of the polymer chains also means that they can slide off each other more easily, which leads to a reduction in viscosity. Thus, the more the molar mass decreases, the lower the viscosity. This is exemplified for polystyrene in Figure 2. In the example shown, the polystyrene was processed on a high-speed twin-screw extruder at different speeds. The higher the speed and, thus, the load on the polystyrene, the lower the viscosity of the processed plastic. This reinforces the correlation between a higher load on the plastic and a greater reduction in viscosity.

The determination of the material degradation can thus be carried out via a reduction in the viscosity. There is an empirical relationship between the weight-average molar mass $M_{w}$ of the polymer and the limiting, respectively, zero viscosity $\eta_{0}$ of the plastic melt. The relationship can be described based on the Mark–Houwink Equation (Equation (1)) for an $\alpha_{\eta }$ of about 3.4 [8,9,10,11,12]:

(1) $$\eta_{0}=K_{\eta }\cdot {\left(\frac{M_{w}}{1\cdot \mathrm{kg}/\mathrm{mol}}\right)}^{\alpha_{\eta }}$$

**Figure 3:**
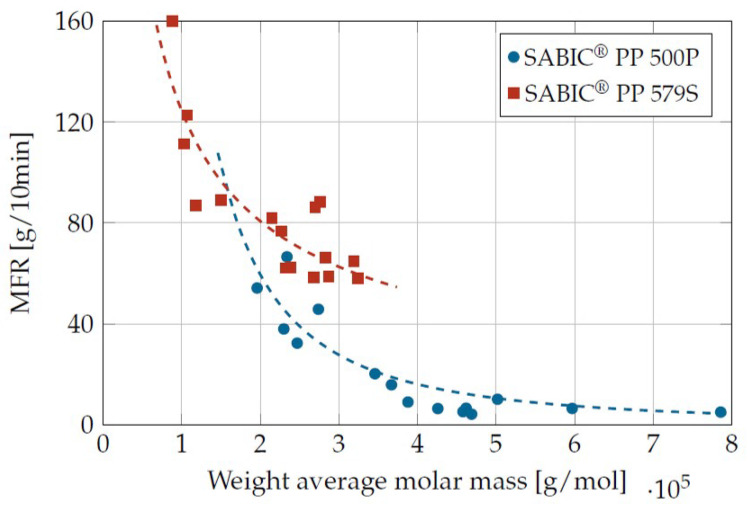
Relationship between MFR value (230 °C/2.16 kg) and molar mass.

The exponential relationship between molar mass and viscosity (here via the MFR value) can be seen in Figure 3 for differently damaged samples. The MFR value is inversely proportional to the zero shear viscosity, so that a high molar mass correlates with a high viscosity and vice versa.

Since it is known that the processing of plastics in a molten state leads to material damage and, thus, to an influence on the material properties, there are many publications in which the degradation behavior during extrusion is investigated, but without clear results. For example, material degradation increases with an increase in back pressure [10] or with a hotter barrel temperature profile [13] and could also be increased by a high filler addition for processing on a twin-screw extruder [14]. Briefly, there is only agreement that material degradation is stronger at higher temperatures than at cooler temperatures [8,10,11,15].

Furthermore, many publications agree that purely thermal degradation below a material-specific temperature can be neglected. Otherwise, the influence of the screw speed for a single-screw extruder is ambiguous. Thus, with increasing speed, degradation increases due to high shear rates [10,16,17] or decreases due to the shorter residence times [11,15]. There is also no agreement as to which influencing factor dominates. In [10], the temperature is mentioned as the most important factor for single-screw extrusion, and in [8] the shear rate is mentioned for the twin-screw extruder.

In many publications, material degradation after multiple extrusions is investigated for both the single-screw and the twin-screw extrusion [12,13,17,18,19,20,21,22,23,24,25,26,27]. Among other things, different screw speeds and screw geometries are investigated here. For example, higher degradation was measured for single-screw extruders at higher shear rates [17] and with increased use of kneading blocks in twin-screw extrusion [18,19]. It is also possible that no difference in viscosity occurs after multiple extrusions, as chain shortening and cross-linking cancel each other out [12].

In [28], a *Processing Degradation Index* (PDI) was even derived, which was based on multiple extrusions on single-screw and twin-screw extruders, which should allow a quantitative prediction about the degradation behavior for the expected occurring loads during processing. Even though the results are close to the application, they can only be transferred to similar processes. Furthermore, in [29], a measurement method is presented which enables an inline and, thus, real-time measurement of the material degradation of polypropylene by using Raman spectroscopy.

In summary, it can only be said that the combination of high temperatures and high shear rates accelerates material degradation [15]. Whether the temperature or the shear rate has the greater influence cannot be answered generally. Since material degradation is, therefore, difficult to measure or even predict, often only a maximum processing temperature is specified for plastics in order to keep material degradation under control [11].

**Figure 4:**
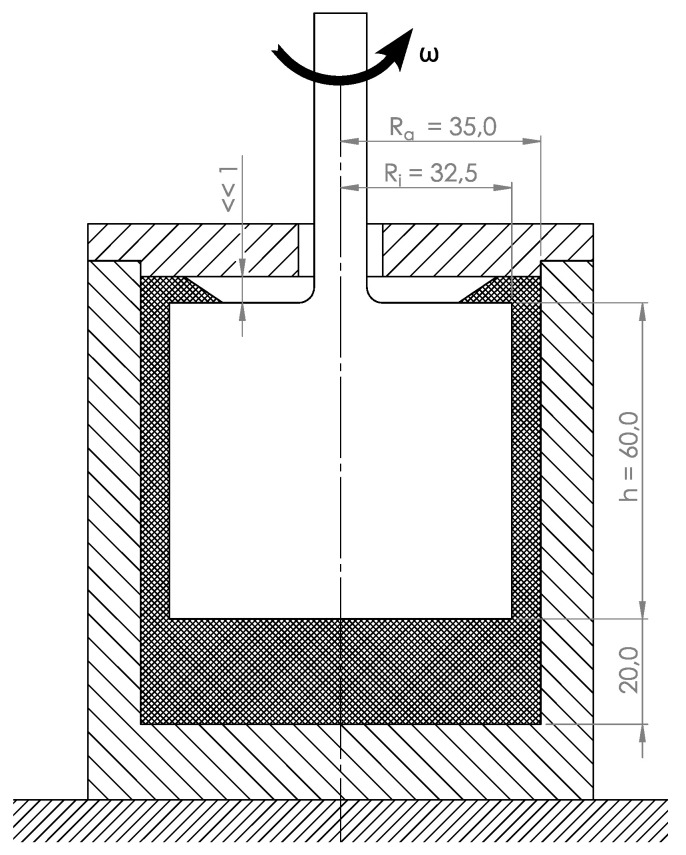
Geometry of the test stand according to Littek [30,31]. Unit: mm.

To address the problem of unpredictable material degradation during processing, material degradation as a function of temperature, shear rate and residence time has already been investigated at Paderborn University [30,31]. Here, plastic samples were damaged in a defined way in a coaxial cylinder system (corresponding to a Searle rheometer, see Figure 4), the samples were taken and the molar mass distribution was determined using gel permeation chromatography.

This was used to derive a prediction model for material degradation. However, the procedure for sample preparation, extraction and measurement is very time-consuming, error-prone and expensive. For this reason, a new test stand and a new measurement methodology have been developed, which are presented in this publication.

## 3. Materials and Methods

The main problem of the test system, according to Littek, is the extraction of the sample and the separate measurement of the molar mass distribution. In order to enable a fast and reproducible measurement of the material degradation, the test stand was revised in such a way that both the damage and the measurement of the material degradation can be carried out without taking a sample. On the one hand, this required a rethought geometry of the cylinder system, so that the damage was as homogeneous as possible in the entire system, and on the other hand, a suitable measurement technique had to be derived. As described in the State of Research, the viscosity of the plastic melt depends on the molar mass. This correlation was used to determine the material degradation no longer on the basis of the costly and time-consuming determination of the molar mass, but on the viscosity of the plastic melt. As can be seen in Figure 2, the viscosity differences, especially at low shear rates, were clearly dependent on the damage history, so that the characterization of the material degradation was based on the reduction in viscosity at the lowest possible shear rate. Due to the selected determination methodology of the material degradation via the reduction in the viscosity, the viscosity was used as a measure of the material degradation. The degree of degradation is thus given by Equation (2). The value does not indicate the reduction in the molar mass, but correlates with it.

(2) $$Degree\;of\;degradation=1-\frac{\eta_{0,damaged}}{\eta_{0}}$$

### 3.1. Measuring Method

The viscosity was calculated from the ratio of shear stress to shear rate:

(3) $$\eta =\frac{\tau }{\dot{\gamma }}$$

**Figure 5:**
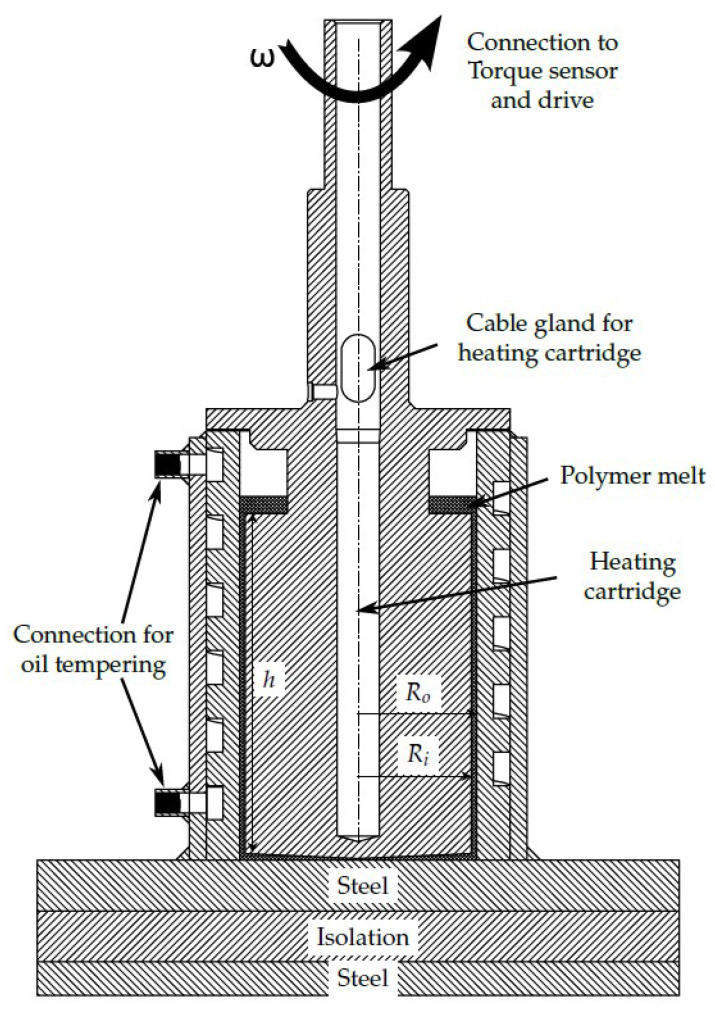
Representation of the geometry of the test stand.

In order to calculate the shear rate and torque as error-free as possible, the geometry of the test system was designed with the help of CFD simulations. This made it possible to reduce to a minimum the boundary influences on shear rate and torque that could not be taken into account analytically. The geometry optimized with CFD simulations is shown in Figure 5.

The gap width ($R_{o}-R_{i}$) was 1.5 mm. By designing the bottom area as a cone-plate system, the same shear rate was also achieved there as in the outer surface and, thus, also the most uniform material degradation possible in the entire system. The known geometry and the torque, measured by means of a torque sensor (Burster precision torque sensor type 8661, linearity deviation < 0.05% f.s.), allowed for the shear rate and shear stress to be calculated:

(4) $$\dot{\gamma }=\omega \cdot \frac{1+\delta^{2}}{\delta^{2}-1}$$

(5) $$\tau =\frac{1+\delta^{2}}{2\cdot \delta^{2}}\cdot \frac{M}{2\cdot \pi \cdot R_{i}^{2}\cdot \left(h+\frac{R_{i}}{3}\right)}$$

(6) $$\delta =\frac{R_{o}}{R_{i}}$$

**Figure 6:**
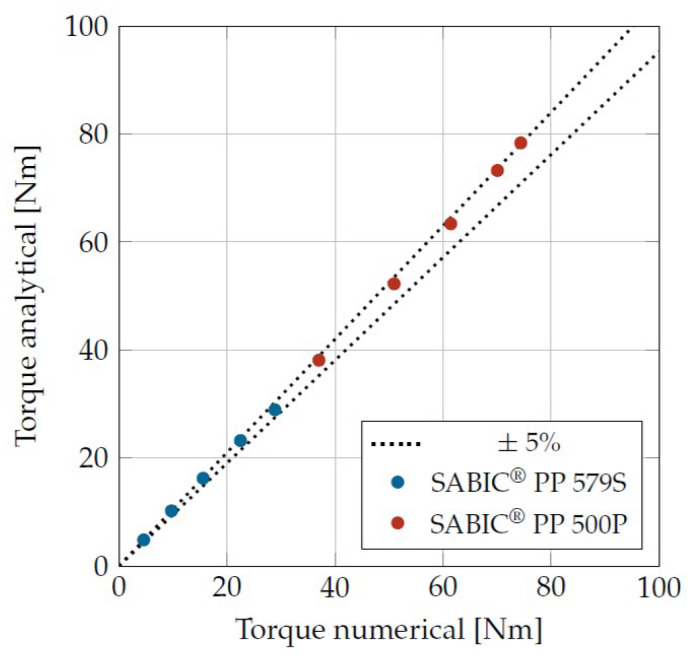
Deviation between the simulated torques and the calculated torques for shear rates of 30, 75, 150, 300, 600 1/s.

The best theoretical accuracy of the viscosity determination can be derived from the CFD simulations. This is shown in Figure 6 for two plastics with different viscosities. The torque is shown, which results simulatively at the inner cylinder under consideration of all occurring boundary effects. This is compared with the torque that results analytically from Equations (4)–(6) under specification of the average viscosity from the CFD simulations. The viscosity was calculated here using the Carreau approach for the shear rate according to Equation (4). The viscosity reduction due to the dissipative temperature increase was also taken into account for the analytical torque. For this purpose, the temperatures from the numerical simulations were not used, as these were not known in the experiment, but the mean temperature was calculated via the FDM method. As in the numerical simulation, isothermal boundary conditions were used. This procedure allowed the expected viscosity to be calculated under the simplifications made and converted into a torque. This resulted in the best accuracy for the analytical correlation between torque and viscosity.

For all operating points investigated, including the high viscosity Sabic PP 500P at a shear rate of 600 1/s, the deviations were within the ±5% interval.

### 3.2. Temperature Control

A prerequisite for the highest possible accuracy in describing the material degradation via the reduction in viscosity is a constant temperature over time. As shown in Figure 5, both cylinders were tempered in different ways. The outer cylinder (stator) was tempered by means of an oil tempering device and a spindle-shaped channel in the outer cylinder. A heating cartridge (630 W) with an integrated thermal sensor was located on the rotation axis of the inner cylinder. To ensure a constant temperature over time, the temperature must also be maintained during operation of the test stand, so while the inner cylinder was rotating. This was achieved by means of sliding contacts. Preliminary tests have shown that the temperature of the inner cylinder corresponds to the set temperature with only slight deviations of less than 2 K.

### 3.3. Measurement Preparation

Before the measurement could start, the plastic had to be in molten form and completely fill the gap in the cylindrical system. It has been shown that the melting procedure has a significant influence on the measurement.

First, the outer cylinder was lowered and a defined volume of plastic granulate (approximately 45 cm^3^) was filled into the outer cylinder. Then, the outer cylinder was pneumatically pressed up with 5 bar pressure so that the plastic melted through the tempered cylinders. For a homogeneous filling of the gap, it has been shown that the inner cylinder must be rotated during the melting process. However, if the rotation is too fast (examined for $\dot{\gamma }\approx$ 10 1/s), significant damage will occur during the melting process. The optimum speed was found to be 0.74 1/min (Motor: 2 rpm, gear ratio 2.7, $\dot{\gamma }$ = 1.77 1/s). As soon as the outer cylinder reached its end position, the plastic was completely molten. The rotation was stopped and, for a homogeneous temperature, another 2 min were waited before the measurement started.

### 3.4. Measuring Procedure

The characterization of the material degradation was conducted via a reduction in the viscosity. The lower the shear rate, and, thus, the closer the flow curve is to the Newtonian viscosity plateau, the greater the viscosity differences that occur. For this reason, the characterization was carried out via the viscosity at the lowest possible shear rate. The lower the shear rate, the better the viscosity differences can be measured. However, the shear rate must be high enough to result in a sufficiently high torque. In experimental investigations, the resulting torques at low shear rates (corresponding to a rotation time of 20, 30 and 40 s) were examined for six different plastics. In each case, the shear rate was selected at which the coefficient of variation of the calculated viscosity was less than 10%. From this, the shear rates for determining the viscosity and, thus, the material degradation were determined according to Table 1.

The measurement of the torque over a complete revolution was an important aspect here in order to avoid angle-dependent measurement errors. Due to slight tensions and eccentricities in the drive train, torques of 0.3 to 0.5 Nm occurred, depending on the angular position of the inner cylinder, caused by the bearing of the drive train. This 0.2 Nm difference already had a significant influence on the calculation of the viscosity. Therefore, the torque was measured over one complete revolution. Furthermore, before each measurement, the torque without plastic, which resulted only from the drive train, was measured and subtracted from the later measurement.

**Figure 7:**
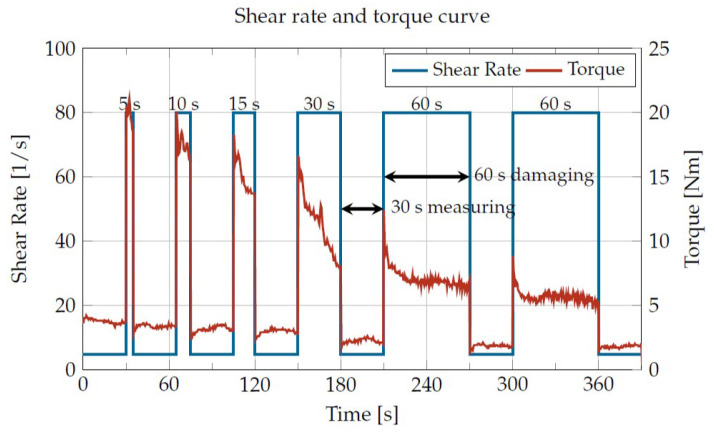
Exemplary course of the shear rate and the torque over time during a measurement for a Borealis PP RD204CF at 230 °C and a damaging shear rate of 80 1/s.

The time sequence of a measurement (Figure 7) was characterized by several measurement and damage phases. The procedure started with a measurement of the viscosity ($\dot{\gamma }$ = 4.78 1/s in the example shown) as a reference for the undamaged plastic, followed by six damage phases ($\dot{\gamma }$ = 80 1/s in the example shown) with subsequent measurement phases for the determination of the viscosity of the damaged plastics. In this way, several damaging times for constant temperature and shear rate can be achieved without sample extraction. In the example shown, cumulative damaging times of 0, 5, 15, 30, 60, 120 and 180 s were obtained. Adding the seven measurement phases of 30 s each resulted in a total duration of 390 s.

### 3.5. Correction of the Measured Data

The measured data were subject to some error influences, which can subsequently be taken into account. For this reason, the measured values were corrected afterwards with regard to three influencing factors.

**Figure 8:**
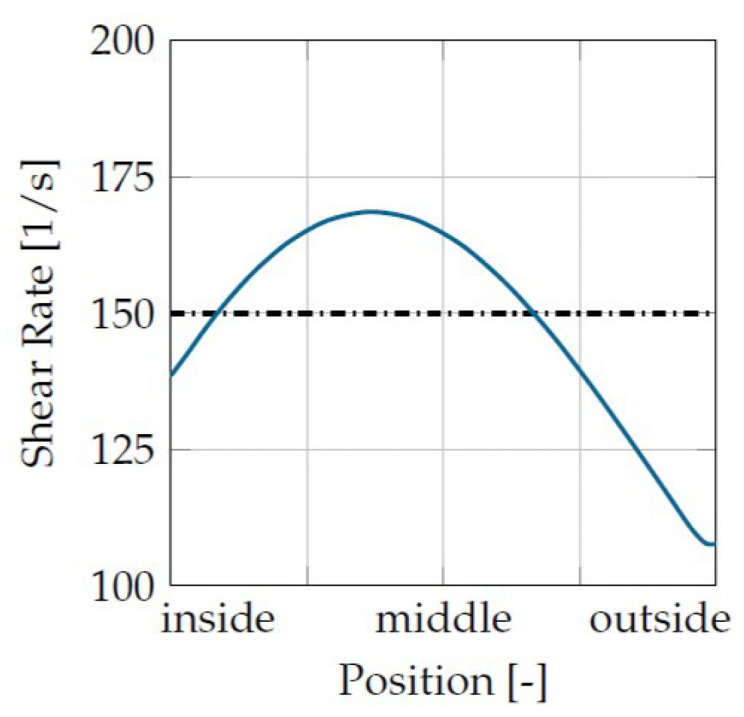
Shear rate distribution.

The first correction concerned the shear rate. The shear rate was not homogeneous in the shear gap due to the shear thinning material behavior. In Figure 8, it can be seen that at a mean shear rate of 150 1/s, the curve at the edge shows a lower shear rate, and, in the middle of the shear gap, a local maximum far above the mean value.

As a result, the plastic in the center of the gap was damaged faster and more severely, so that there was also an above-average reduction in viscosity. It was assumed that this low-viscosity layer behaved like a “sliding film”, which reduced the torque in the measurement and, thus, the degradation behavior was overestimated.

This was countered by assuming that the shear rate in the evaluation was higher than the mean set shear rate. This was performed by calculating the shear rate in the evaluation using Equation (7). This is the wall shear rate of a Couette flow.

(7) $${\dot{\gamma }}_{w}=\frac{\left(\frac{2}{n}\right)\cdot \omega }{1-k^{\frac{2}{n}}}\;\;\;with\;\;\;k=\frac{R_{i}}{R_{o}}$$

By assuming a higher shear rate, the overestimation of material degradation due to the inhomogeneous shear rate was counteracted. The comparison of the mean set shear rate to the corrected shear rate is shown in Table 2 for a Sabic PP 500P (power-law flow exponent n = 0.28 according to Ostwald–de Waele).

The second correction concerned the temperature in the shear gap. The dissipation in the shear gap resulted in a non-measurable inhomogeneous temperature distribution of the plastic with a maximum near the center of the shear gap and approximately the set temperature at the tempered cylinders. On the one hand, the temperature maximum in the center of the gap supported the effect of the “sliding film”; on the other hand, the increased temperature increased the overall degradation rate in the shear gap. Therefore, the increase in temperature was also taken into account and the temperature of the evaluation was set to the average temperature in the shear gap under damage conditions. Although this cannot be measured, it can be derived using CFD (computational fluid dynamics) or FDM simulations (finite difference method), for example. According to CFD and FDM simulations, a steady-state temperature distribution already occurs after a few seconds, so that only the steady-state temperature can be considered for the evaluation.

For the evaluation, the calculation of the temperature by means of the FDM was used due to the fast calculation time. The calculation was performed assuming isothermal inner and outer cylinders and dissipation as the source term. The dissipation can be calculated according to the Equation (8).

(8) $${\dot{q}}_{Dissipation}=\eta \cdot {\dot{\gamma }}^{2}$$

The temperature calculation using FDM shows very good agreement with the temperature simulation using CFD. However, FDM has the advantage of a very fast calculation. Furthermore, only the thermal material properties are needed, since shear rate and viscosity are already derived from the measured data. In Table 3, for example, the set and calculated temperatures are compared by means of the FDM calculation. The temperature differences were 0.1 K at low shear rates and up to 7 K at shear rates of 150 1/s.

**Figure 9:**
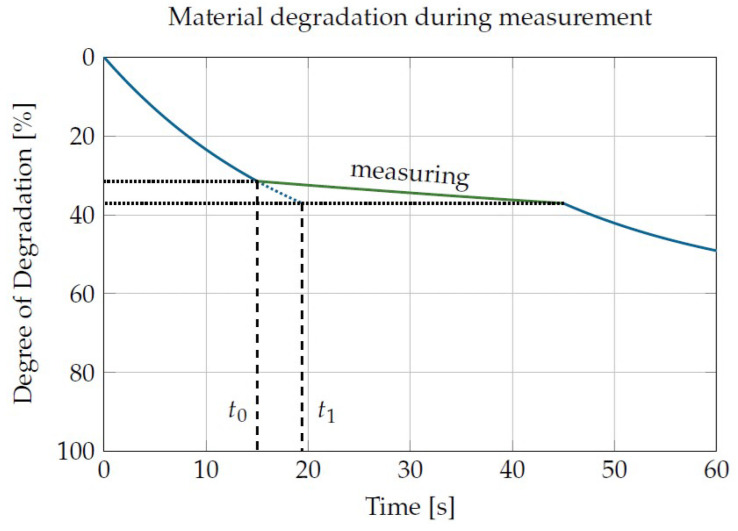
Consideration of material damage during the measurement phases.

The third correction dealt with the material degradation that took place during the measurement phases. Since 7 measurement phases of 20 to 40 s each were carried out in the presented measurement sequence, the material degradation must be taken into account, even if the load was low during this time. An exemplary course of the degree of degradation is shown in Figure 9. After 15 s of high load ($t_{0}$), a 30 s measurement phase with low load follows. The degradation level after the measurement phase is slightly higher than the degradation level before the measurement phase. In order to take into account the reduction in the degree of degradation caused by the measurement in the evaluation, the degree of degradation after the measurement phase is converted into a theoretical time ($t_{1}$) at which the degree of degradation would have been reached under damage conditions. Instead of calculating the degradation during the measurement from the measurement data, the damage times were corrected in this way. This had the advantage of a data set for constant temperature and shear rate results for each sample, which is mathematically much easier to handle than a data set with different loading levels.

A prerequisite for this correction, however, is that the degradation during the measurement phase can be calculated. For this purpose, the measured data, corrected for shear rate and temperature, were described by means of a mathematical Equation (see Section 3.7), so that a prediction was possible for any load, and, thus, also for the influence of the measurement phases on material degradation. However, since the mathematical description of the measurement data depends on this correction and this correction also depends on the parameters of the mathematical description, an iterative procedure was necessary.

From the correction made, exemplary thoretic damage times resulted according to Table 4. For example, the adjusted damage times of the Sabic PP 500P increased from 5 s to 6.48 s, but also from 180 s to 254.82 s.

**Figure 10:**
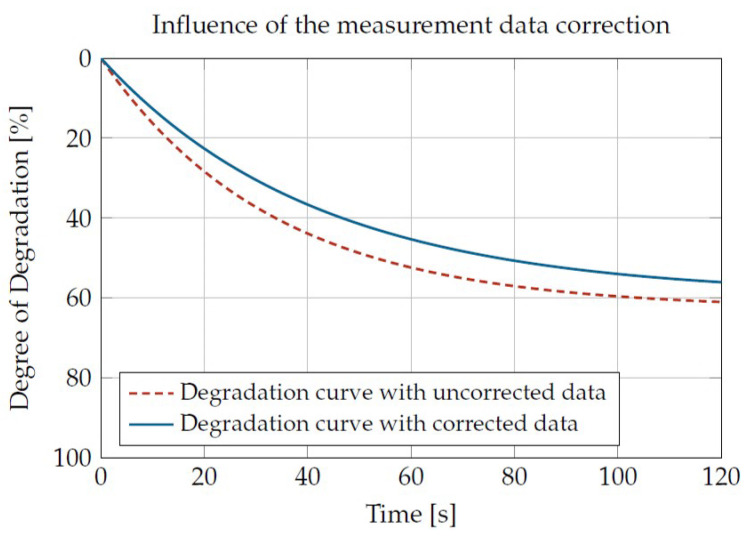
Influence of the correction (Sabic PP 500P at 220 °C and 50 1/s).

All three presented corrections led to a slower degradation speed compared with the uncorrected data. This is shown as an example in Figure 10. The expected degradation process without and with correction of the measured data for an identical load is shown.

### 3.6. Repeatability

The repeatability is of crucial importance for material characterization. Only if reproducible results can be generated is it a suitable measurement method. For the evaluation of the repeatability, five characterizations were performed for a Sabic PP 500P. The first three characterizations were performed by a test stand operator in February and March 2022, each one week apart. A fourth and fifth characterization were performed in September 2022 by two additional test stand operators under the same ambient conditions. However, the influence of ambient conditions is not expected to be significant.

**Figure 11:**
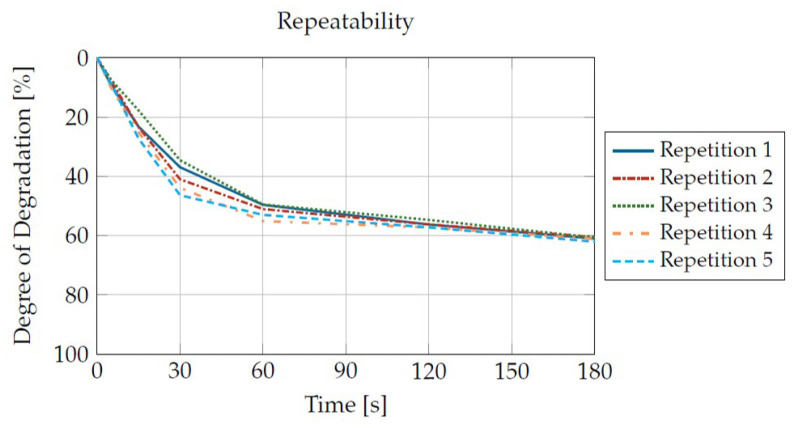
Repeatability of the test stand and the measurement procedure.

With a mean deviation of 2.25% of all measured values related to the mean value of all measurements, good repeatability can be attested. This can be seen as an example in Figure 11. Here, the degradation curves for a Sabic PP 500P at 235 °C and a shear rate of 80 1/s are shown. Although slight differences can be seen, the curves are very similar.

### 3.7. Mathematical Description of the Data

To use the measured and corrected data as a prediction model, a mathematical description for any occurring loads was needed. For this reason, a mathematical description according to Equation (9) was derived. This equation represents an exponential decay function and reflects the reduction in viscosity due to the influencing variables’ shear rate, temperature and residence time.

(9) $$\frac{\eta_{0,damaged}}{\eta_{0}}=\left(1-\eta_{\infty }^{*}\right)\cdot \exp\left[-\left({\left(\frac{T}{K_{T}}\right)}^{n_{T}}+{\left(\frac{\dot{\gamma }}{K_{\dot{\gamma }}}\right)}^{n_{\dot{\gamma }}}\right)\cdot \frac{t}{1s}\right]+\eta_{\infty }^{*}$$

The equation requires five material parameters that characterize the degradation behavior. The factors $K_{T}$ and $n_{T}$ describe the degradation behavior by temperature influence and the factors $K_{\dot{\gamma }}$ and $n_{\dot{\gamma }}$ by shear rate, each as a function of time *t*. The parameter $\eta_{\infty }^{*}$ describes the ratio of the viscosity of the damaged to the undamaged sample for an infinite time. The value thus lies between 0 and 1.

**Figure 12:**
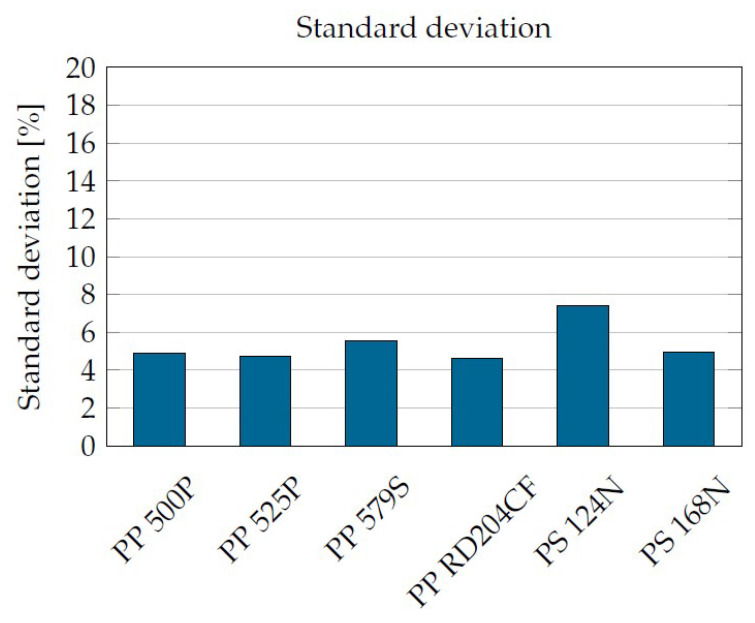
Standard deviation of the equation to the corrected measured data.

The material parameters were derived via variance minimization with respect to all measured values. The resulting standard deviations with best-possible regression for six different plastics are shown in Figure 12.

**Figure 13:**
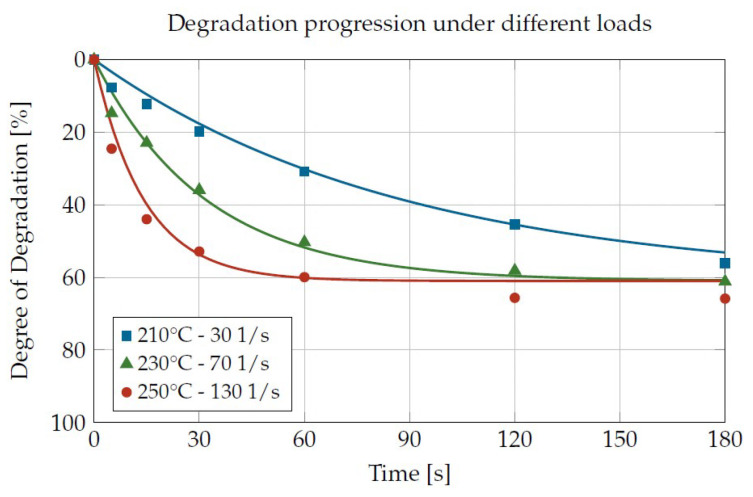
Agreement of the mathematical description with the measurement data for the PP RD204CF.

With standard deviations of less than 8%, a high accuracy can be achieved in the description of the measured data. This can be seen in Figure 13. Furthermore, extrapolation to even lower and even higher loadings is possible without generating physically implausible values. The use of the material characterization outside of the measured values is to be used here naturally; nevertheless, they are only used with caution.

## 4. Results

With the test stand presented, the measurement methodology used and the mathematical description provided, plastics can be characterized in terms of their degradation behavior as a function of temperature, shear rate and residence time. The five material parameters according to Equation (9) for the six investigated plastics are shown in Table 5.

**Figure 14:**
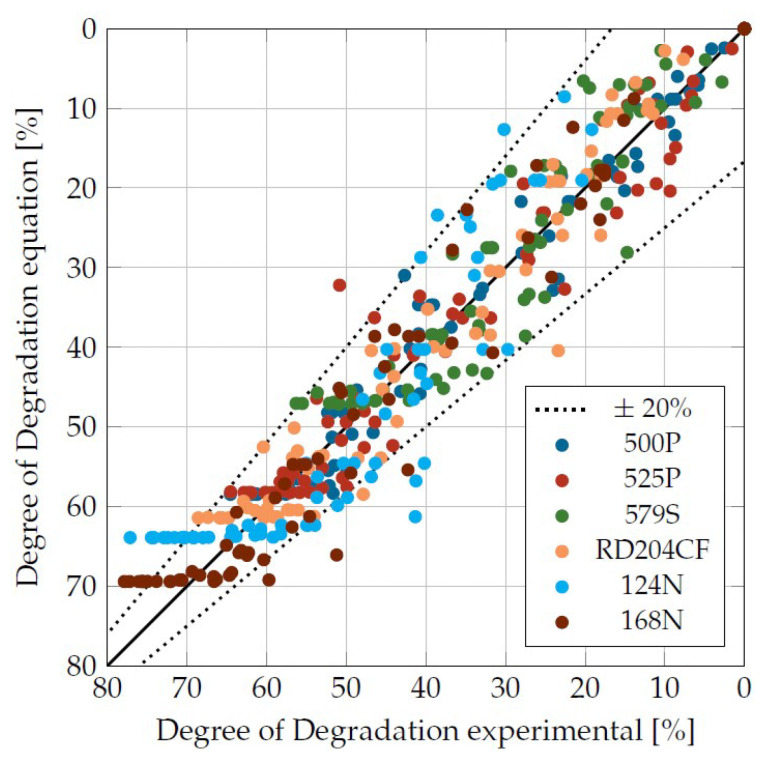
Agreement between measured and predicted values.

From the experimental two-dimensional CCD test plan with 11 test points (3 times the central point, 4 corner points, 4 star points. Six different damage times each), 66 values for material degradation per plastic were obtained. Figure 14 shows a comparison for the six different plastics from the experimentally determined viscosity degradation to the calculated viscosity degradation according to Equation (9).

The dashed lines represent the ±20% interval. The majority of the 396 total test points were located within the interval, so that the accuracy of the mathematical description can be considered satisfactory.

**Figure 15:**
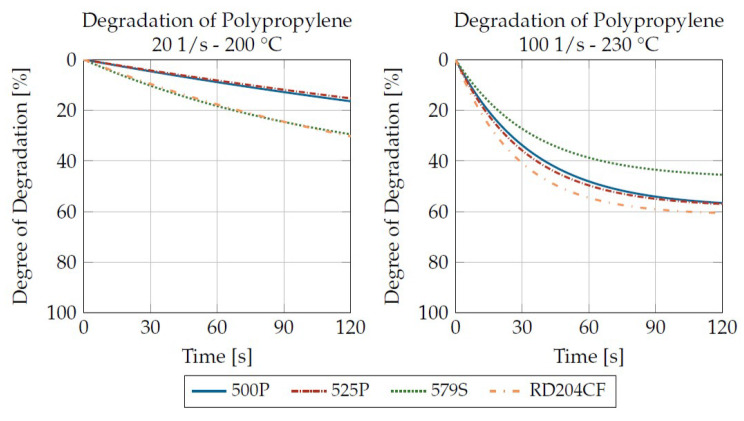
Comparison of the degradation behavior of the four polypropylenes under identical loads.

By parameterizing the material degradation via the influencing factors’ shear rate, temperature and residence time, a statement can now be made, depending on the material, as to which influencing factor has the greatest effect on the material degradation. It is not possible to make a general statement here, as each of the plastics investigated has a different behavior. Figure 15 shows the degradation curves for the four polypropylenes investigated under identical loadings. It can be seen that at low loadings, the degree of degradation only decreases slowly. At higher loads, the viscosity decreases much more rapidly and approaches a final value after the 120-s residence time shown.

The Sabic PP 500P and 525P (MFI 3.1 and 3.0 g/10 min (230 °C/2.16 kg)) exhibit a very similar rheological property profile. This is also reflected in a very similar degradation behavior for both loads presented. It can therefore be assumed, but not proven due to a lack of sufficiently large data, that plastics of the same polymer type with basically very similar properties also exhibit similar degradation behaviors. In contrast, there are the Sabic PP 579S (MFI 47 g/10 min (230 °C/2.16 kg)) and the Borealis RD204CF (MFI 8 g/10 min (230 °C/2.16 kg)). At low loading, the degradation rate is almost identical. At the high load, the degradation rate is much greater for the RD204CF and the low viscosity 579S degrades much slower than the other plastics tested. This can be attributed to the fundamentally lower viscosity and molar mass of the 579S.

Thanks to the mathematical description, the degradation behavior of the characterized plastics can subsequently be used to predict the material degradation with known influencing variables (shear rate, temperature and residence time). At this point, two examples of applications will be presented.

**Figure 16:**
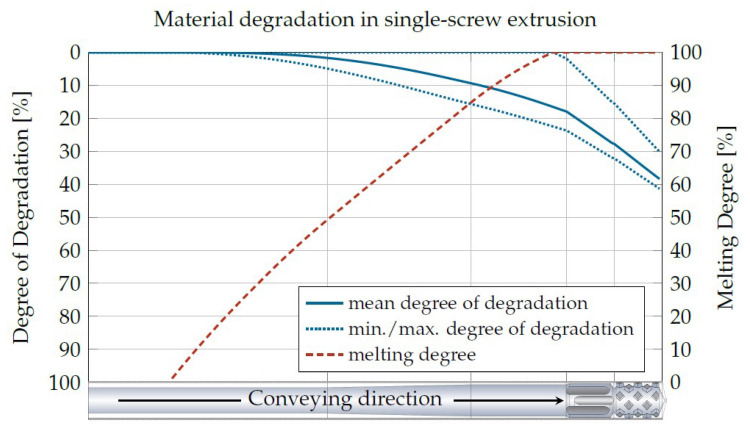
Degree of degradation based on REX data.

One way to predict material degradation in single-screw extrusion is to use REX, a program for the analytical simulation of single-screw extruders. By means of analytical equations, mass throughput, residence time, shear rate, temperature profile and melting profile, among others, are calculated within a few seconds. Based on these data, a calculation of the material degradation and, thus, the reduction in viscosity can be made. An example of degradation, calculated on the basis of REX, can be seen in Figure 16.

A second possibility to use the material characterization as a prediction model are numerical simulations. In principle, two different methods are available here. One possibility is the transient numerical simulation, in which each cell of the computational grid receives the value “degree of degradation” as a user-defined scalar and the degree of degradation is recalculated after each time step by means of a user-defined function. With this procedure, a coupling with the viscosity is also possible. However, the long calculation times are a disadvantage of transient simulations.

A faster calculation is achieved with the second option of steady-state numerical simulation. Since no discrete time steps are calculated here, the simulation is, on the one hand, significantly faster, but, on the other hand, the influencing variable time for the degradation model is missing. At this point, the degree of degradation can only be determined in post-processing by calculating streamlines. Streamlines correspond to the flow course of an infitisimal small particle along the velocity field of the simulation area. Thus, for each streamline, a course with the residence time as well as the locally present temperatures and shear rates is obtained, so that the calculation of the progressive material degradation is possible for each individual streamline. The degree of degradation at the flow outlet can be averaged over as many streamlines as possible at this point.

## 5. Discussion

The degradation behavior of thermoplastics can be characterized with the presented measurement methodology and data preparation. However, there are limitations in the measurement accuracy and the selection of the polymers that can be investigated.

The quality of the generated data depends not only on the measuring accuracy of the torque sensor, but also on the boundary influences of the generated flow and the inhomogeneity of the flow in the test system. The inhomogeneities concerning the shear rate distribution, the temperature increase due to dissipation and, furthermore, the influence of the measurement of the material degradation on the damage of the specimen can be taken into account to some extent by the presented corrections. How accurate these corrections are cannot be answered, since no reference values can be measured for validation. The evaluation of the accuracy of the measurement data must therefore always take into account the measurement system as well as the measurement data correction.

A disadvantage of the measurement method is that only plastics with simple degradation behavior can be investigated. Plastics that are prone to hydrolysis, post-polymerization, branching or crosslinking cannot be described by their viscosity reduction, since processing of the plastic samples here can also lead to an increase in viscosity. In the case of hydrolysis susceptibility in plastics synthesized via polycondensation, the degradation behavior also depends on the moisture content in the plastic, which cannot be taken into account with the current equation for describing material degradation.

A further limitation exists with very high-viscosity plastics ($\eta_{0}$ >> 10,000), since here the driving servo motor (standstill torque of 69 Nm) is not sufficiently strong enough to achieve the required damage speeds.

Likewise, the centricity of the test system cylinders has a significant influence on the measurement results. Due to the narrow 1.5 mm gap, even small eccentricities have a great influence on the homogeneity of the gap width. An eccentricity can cause the test stand to vibrate and material can also be pushed out of the gap. This not only leads to inhomogeneous damage, but also to a reduction in the torque, so that an evaluation can no longer be carried out. However, if this happens unnoticed at high speeds, it results in erroneous measured values.

## 6. Conclusions

The prediction of the material degradation of plastics is not state-of-the-art. Although there are studies that investigate material degradation experimentally and provide conclusions on this, these findings are rarely transferable to other processes and, thus, are rarely usable for predicting material degradation, for example in the context of screw design. Furthermore, some publications contradict each other regarding the significant influencing parameters or the observed tendencies. In order to enable a prediction of the material degradation already in the screw design, a test stand for the material characterization as well as a mathematical description of the degradation behavior are described within this publication. In order to enable a basically fast material characterization, the reduction in the zero viscosity is used as a measure for the material degradation. This has the advantage of a fast and simple measurement. Likewise, a one-step procedure can thus be implemented, since targeted damage of the plastic samples as well as the measurement of the viscosity of the samples can be carried out within the same test stand without having to extract samples.

To calculate the viscosity as accurately as possible, a suitable cylindrical geometry of the test stand was derived by means of numerical simulations. The measurement procedure and subsequent data processing are also described, so that an accurate and, at the same time, reproducible measurement is possible. With the test stand and measuring procedure presented, a plastic can be examined within a few hours for its degradation behavior as a function of temperature, shear rate and residence time. In order to further develop a prediction model from the data obtained, a mathematical model is presented to describe the degradation behavior. By means of the relatively accurate mathematical description of the degradation behavior, prediction models are presented as possible applications for analytical extruder calculations as well as for numerical simulations. Thus, in connection with the measuring method, the test stand provides a possibility for material characterization as well as the use of the generated data as a prediction model. This makes it possible to consider the expected material degradation, for example, as early as the screw design stage, so that in the future it will be possible to design screws with material-conserving process behaviors.

## Figures and Tables

**Table 1:** Shear rates for the determination of viscosity. Given zero shear viscosities for 230 °C.

Plastic	Zero Shear Viscosity	Shear Rate
Styrolution^®^ PS 168N	4190 Pa·s	3.54 1/s
SABIC^®^ PP 500P	4110 Pa·s	3.54 1/s
SABIC^®^ PP 525P	3520 Pa·s	3.54 1/s
Borelis PP RD204CF	1030 Pa·s	4.78 1/s
Styrolution^®^ PS 124N	960 Pa·s	4.78 1/s
SABIC^®^ PP 579S	220 Pa·s	7.27 1/s

**Table 2:** Comparison of the corrected shear rate to the mean shear rate for the SABIC^®^ PP 500P.

Set Mean Shear Rate [1/s]	Assumed Shear Rate [1/s]
10	11.64
30	34.92
80	93.12
130	151.32
150	174.60

**Table 3:** Comparison of the corrected temperature to the set temperature for a SABIC^®^ PP 500P.

Set Temperature [°C]	Calculated Temperature [°C]	at Shear Rate [1/s]
210	213.3	80
217	217.8	30
217	223.6	130
235	235.1	10
235	237.9	30
235	241.7	150
253	253.5	30
253	257.4	130
260	261.9	80

**Table 4:** Example comparison of the corrected times to the set times for a SABIC^®^ PP 500P at 235 °C and 80 1/s.

Set Time [s]	Corrected Time [s]
0	0.00
5	6.48
15	17.96
30	34.44
60	65.93
120	127.41
180	254.82

**Table 5:** Material parameters of the investigated plastics and resulting standard deviation σ.

Manufacturer	Designation	$K_{T}$ [°C]	$n_{T}$ [-]	$K_{\dot{\gamma }}$ [1/s]	$n_{\dot{\gamma }}$ [-]	$\eta_{\infty }^{*}$ [-]	σ [%]
SABIC^®^	500P	345.7	15.29	1171.4	1.47	0.415	4.92
SABIC^®^	525P	355.3	13.75	882.3	1.62	0.417	4.75
SABIC^®^	579S	1617.0	3.15	6989.3	0.85	0.531	5.55
Borealis	RD204CF	344.8	15.41	1861.1	1.15	0.386	4.64
Styrolution^®^	168N	17,326.2	1.22	5749.3	0.81	0.306	4.97
Styrolution^®^	124N	761.5	40.36	8740.6	0.63	0.361	7.42

## Data Availability

Not applicable.

## Abbreviations

The following abbreviations are used in this manuscript:

*h*	Height of the shear gap [m]
*k*	Ratio of inner to outer radius [-]
$K_{T}$	Temperature sensitivity [°C]
$K_{\dot{\gamma }}$	Shear sensitivity [1/s]
$K_{\eta }$	Material parameter [Pa·s]
*M*	Torque [Nm]
$M_{w}$	Weight average molar mass [kg/mol]
*n*	Flow exponent [-]
$n_{\dot{\gamma }}$	Shear sensitivity exponent [-]
$n_{T}$	Temperature sensitivity exponent [-]
${\dot{q}}_{Dissipation}$	Specific dissipation [W/m^3^]
rpm	Revolutions per minute
$R_{i}$	Inner radius of the shearing gap [m]
$R_{o}$	Outer radius of the shearing gap [m]
$\alpha_{\eta }$	Material parameter [-]
$\dot{\gamma }$	Shear rate [1/s]
δ	Ratio $R_{o}$ to $R_{i}$ [-]
η	Viscosity [Pa·s]
$\eta_{0}$	Zero shear viscosity [Pa·s]
$\eta_{0,damaged}$	Zero shear viscosity of the damaged sample [Pa·s]
$\eta_{\infty }^{*}$	Relative final viscosity [-]
τ	Shear stress [Pa]
ω	Angular velocity [rad/s]
